# Electrolyte Effect on Electrocatalytic CO_2_ Reduction

**DOI:** 10.3390/nano15090648

**Published:** 2025-04-25

**Authors:** Jiandong Zhang, Ziliang Zhang, Tianye Chen, Jiayi Zhang, Yu Zhang

**Affiliations:** 1 College of Civil Engineering and Architecture, Zhejiang University, Hangzhou 310058, China; 2 School of Electro-Mechanical Engineering, Guangdong University of Technology, Guangzhou 510006, China; 3122000399@mail2.gdut.edu.cn; 3 Faculty of Mechanical Engineering and Mechanics, Ningbo University, Ningbo 315211, China; 236001862@nbu.edu.cn (T.C.); 236003994@nbu.edu.cn (J.Z.)

**Keywords:** electrocatalytic CO_2_ reduction, pH effects, cation effects, anion effects

## Abstract

Electrocatalytic CO_2_ reduction reaction shows great potential for converting CO_2_ into high-value chemicals and fuels at normal temperature and pressure, combating climate change and achieving carbon neutrality goals. However, the complex reaction pathways involve the transfer of multiple electrons and protons, resulting in poor product selectivity, and the existence of competitive hydrogen evolution reactions further increases the associated difficulties. This review illustrates the research progress on the micro mechanism of electrocatalytic CO_2_ reduction reaction in the electrolyte environment in recent years. The reaction pathways of the products, pH effects, cation effects and anion effects were systematically summarized. Additionally, further challenges and difficulties were also pointed out. Thus, this review provides a theoretical basis and future research direction for improving the efficiency and selectivity of electrocatalytic CO_2_ reduction reaction.

## 1. Introduction

Electrocatalytic CO_2_ reduction reaction (CO_2_RR) has exhibited immense potential as a promising solution to address climate change by converting CO_2_ into high-value chemicals and fuels, a process that can be carried out under ambient temperature and pressure with adjustable reactants and driven by renewable energy sources such as wind and solar power [1,2,3,4]. Nevertheless, the reduction pathways are intricate and involve multiple electron (i.e., e^−^) and proton (i.e., H^+^) transfers, leading to low selectivity. Additionally, the competing hydrogen evolution reaction (HER) presents a significant challenge in realizing high local current density and faradaic efficiency (FE) [5,6]. Thus, the development of efficient electrocatalytic reduction technologies is of great practical significance.

**Figure 1:**
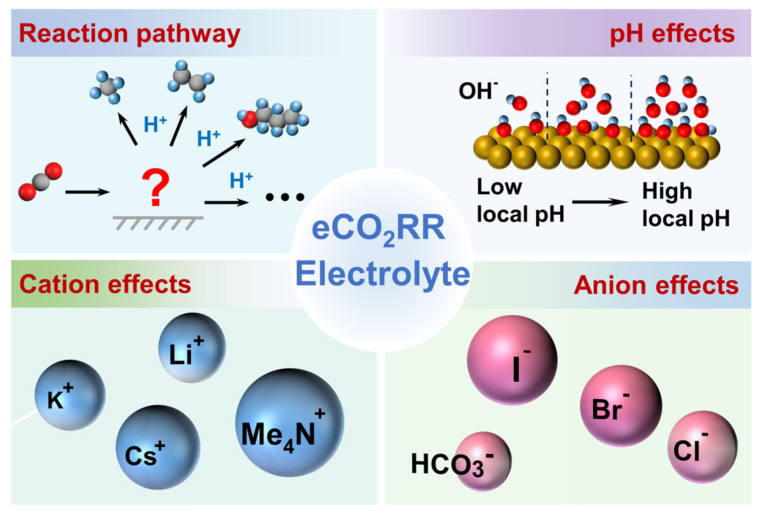
The overview diagram of the content of the review. The variation of the reaction pathway is first summarized to understand how the electrolyte influences product selectivity. Then, the effects of pH, cations and anions are reviewed. Note, the red “?” represents the possible reaction pathways.

The electrocatalytic CO_2_RR occurs within the electrical double layer (EDL) at the electrode–electrolyte interface (EEI), so its efficiency relies not only on the electrode materials (i.e., catalysts) but also on the electrolyte. Previous studies have primarily concentrated on the optimization of catalysts, such as enhancing catalytic performance through morphology and facet engineering [7,8], vacancy steering [9], doping modification [10,11], alloying [12] and single-atom sites [13,14,15]. These strategies increase the number of active sites on the catalyst surface, alter the material electronic structure and local charge polarization or realize synergistic effects between multiple components, optimizing the adsorption or desorption reactions of intermediates and charge transfer process. Despite advancements, only Cu-based catalysts have been proven to reduce CO_2_ to multi-carbon products, but the activity remains low [16,17,18]. Consequently, merely depending on catalyst optimization may not fully resolve the challenges faced by CO_2_RR. In this context, as an indispensable part of the reaction, the electrolyte has also gained much attention, not only providing protons but also directly affecting the formation of intermediates and the reaction pathway [19,20,21,22,23]. As outlined in Figure 1, the variation of the reaction pathway is first summarized to understand how the electrolyte influences product selectivity, then the electrolyte’s effects are extensively studied, including pH effects [24,25,26], cation effects [27,28,29,30,31], and anion effects [32,33,34]. Nevertheless, despite immense achievements, the specific effect may differ across studies, resulting in limited consensus. For instance, due to the coexistence of cations and anions at the EEI, multi-interactions overcomplicate related research. Buffer ions are generally introduced to control pH, which leads to confusion between the effects of pH and ions, making it challenging to isolate their individual contributions [23]. Thus, this field lacks a critical and systemic synthesis to summary conflicting observations and the underlying causes of discrepancies in electrolyte effects.

Unlike previous reviews that primarily catalog electrolyte effects [35,36,37], this work critically examines the contradictions among existing studies and identifies key factors (e.g., interfacial field screening, buffer-induced artifacts, and ion cooperativity) that lead to divergent conclusions. By establishing a mechanistic framework that disentangles these complexities, this work provides not only a unified perspective but also practical guidelines for future experimental design—a step toward resolving long-standing debates in CO_2_RR electrolyte engineering.

## 2. Reaction Pathway

**Figure 2:**
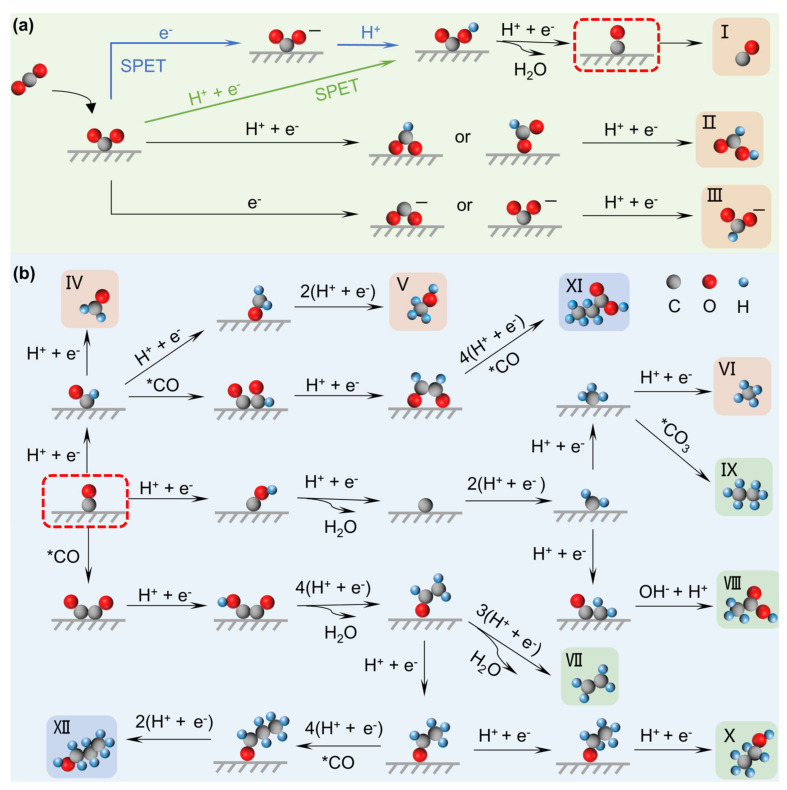
(**a**) Two pathways of electron transfer, and (**b**) overview of reaction pathways for CO_2_RR towards different products [42]. Copyright 2024, Elsevier.

Proton-coupled electron transfer (PCET) processes occur in CO_2_RR,* and the number of electrons involved in the reaction may vary depending on the catalyst and reaction conditions, typically ranging from 2e^−^, 4e^−^, 6e^−^, and 8e^−^ to 12e^−^ or even more [23]. The difference in electron number directly determines the type of reduced products [18], as shown in Table 1. The products of CO_2_RR include carbon monoxide (CO), formic acid (HCOOH) and formate (HCOO^−^), oxalic acid (H_2_C_2_O_4_) and oxalate (C_2_O_4_^2−^), methane (CH_4_), formaldehyde (CHOH), methanol (CH_3_OH), ethylene (C_2_H_4_) and ethanol (C_2_H_5_OH) [7,38,39,40,41]. All products begin with the adsorption of CO_2_ on the catalyst surface to *CO_2_ intermediate (* indicates an active site), and the configuration of *CO_2_ largely determines the reaction pathway. As shown in Figure 2a, the proton–electron transfer occurs via two pathways [42]. The blue arrow represents the sequential proton–electron transfer (SPET), where the *CO_2_ first accepts an electron to form *CO_2_^−^ with the carbon coordinated to the catalyst, and then accepts a proton to form *COOH (a key precursor to *CO). The green arrow represents the concerted proton–electron transfer (CPET), where the *CO_2_ directly gains both a proton and an electron to form *COOH [43]. *CO can desorb from the catalyst surface to generate CO (Product I), so the pathway can be described as follows: *CO_2_→*COOH→*CO→CO. Apart from *COOH, based on the *CO_2_ configuration, CO_2_ can also be hydrogenated to HCOO*, ultimately generating HCOOH (Product II) or HCOO^−^ (Product III) [44]. Other product pathways are depicted in Figure 2b. HCHO (Product IV), CH_3_OH (Product V) and CH_4_ (Product VI) are also common C_1_ products, which involve 4e^−^, 6e^−^, and 8e^−^ electron transfer. Intricately, competing reactions exist between different products, such as the intermediates for HCHO and CH_3_OH being *CHO. The intermediates also vary depending on the catalyst surface and reaction conditions. For instance, Shi et al. [42] described that *COH can directly dehydrate to *C, then be further hydrogenated to CH_4_. However, other studies suggest that *COH first combines with H to *CHOH, which then undergoes dehydration to form *CH, followed by proton coupling to generate CH_4_ [45,46].

Multi-carbon products involve more proton–electron transfer and follow complex reaction pathways. Figure 2b summarizes a series of possible multi-carbon products, including C_2_H_4_, CH_3_CH_2_OH, ethane (C_2_H_6_), ethylene glycol (C_2_H_6_O_2_), acetic acid (CH_3_COOH) and propanol (C_3_H_7_OH) [42,46,47,48,49,50]. *CO is considered a key intermediate for C_2_ products, and the C-C coupling is the RLS [51]. The widely accepted pathway is that *CO undergoes C-C coupling to generate C_2_ products (e.g., *CO + *CO→*OCCO). Taking the generation pathway of C_2_H_4_ as an example, Qiu et al. [52] proposed that C_2_H_4_ formation requires combining CO and *CHO to create *COCHO, then the two carbon atoms hydrogenate and deoxygenate to *CCH, which is further hydrogenated to form C_2_H_4_. However, an alternative view is that *CO and *CO directly undergo C-C coupling to form *OCCO [53]. In this pathway, the coupled C atoms undergo hydrogenation and deoxygenation to form *CCO, which then proceeds through PCET steps to generate CHCO, CHCHO, CH_2_CHO, and ultimately C_2_H_4_. Thus, exploring the universality of reaction pathways remains a critical focus, and studies on the mechanisms of multi-carbon products are still limited, requiring further exploration and validation.

## 3. pH Effects

The selectivity of CO_2_RR is significantly influenced by the electrolyte pH, with the main difference between acidic and neutral/alkaline electrolytes being the proton source. As shown in Table 1, in acidic media, hydrated hydrogen ions (H_3_O^+^) act as the proton source, while water molecules serve as proton donors in neutral/alkaline electrolytes [54,55]. Typically, CO_2_RR is conducted in neutral or alkaline electrolytes, since a higher pH helps suppress the competing HER [26,56]. However, some studies indicate that HER at the reversible hydrogen electrode (RHE) is independent of pH, as it can be driven by the reduction of water molecules [18,57,58]. In an alkaline environment, CO_2_ can not only be directly reduced on the electrode surface but may also react with OH^−^ ions to form carbonates (CO_3_^2−^) or bicarbonates (HCO_3_^−^). Specifically, CO_2_ + 2OH^−^→CO_3_^2−^ + H_2_O and CO_2_ + OH^−^→HCO_3_^−^, both of which cannot directly participate in the reduction reaction, leading to carbon loss and low conversion efficiency [59,60,61].

To address the above issue, acidic electrolytes have become an emerging area of interest in CO_2_RR, effectively avoiding the CO_3_^2−^ and HCO_3_^−^, while a higher H^+^ concentration makes HER more kinetically favorable. To suppress HER, researchers have proposed various strategies. For example, Bondue et al. [62] studied CO_2_RR on gold electrodes under mild acidic conditions and found that the rates of CO and OH^−^ generation must be sufficiently high to effectively suppress HER. Huang et al. [24] reported that adding high concentrations of alkaline metal cations (AMCs) to acidic electrolytes can enhance the local pH or electric field, effectively increasing the current density. Additionally, active site engineering has been applied to adjust the interaction between key intermediates like *COOH, *OCOH, and *OCCO with the catalyst surface. However, the stability of CO_2_RR in acidic electrolytes is much lower than that in basic ones, as the higher H^+^ concentration and the lack of HCO_3_^−^/CO_3_^2−^ ions hinder stable progression. Meanwhile, the acidic environment leads to the dissolution of many metal or metal oxide catalysts, and the degradation is uncontrollable [63,64,65,66,67].

**Figure 3:**
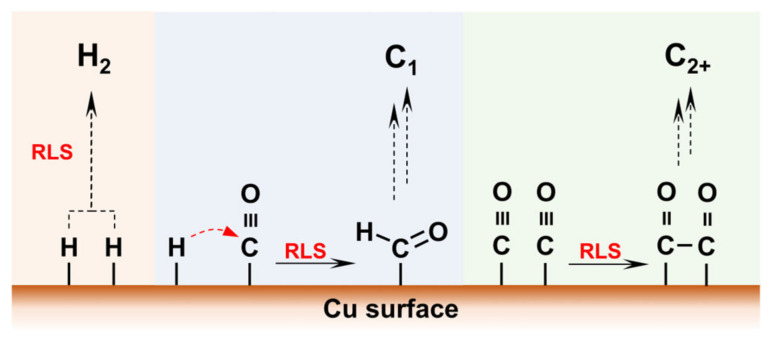
The mechanism model for C_1_, C_2+_ and H_2_ evolution [35]. Copyright 2022, American Chemical Society.

Apart from bulk pH, the local pH at the EEI significantly affects catalytic selectivity and product distribution. Hori et al. [68] were the first to propose that local pH could alter the reaction pathways of CO_2_RR. The local pH is related to the formation of key intermediates, primarily because it can determine proton transfer or rate-limiting steps (RLSs), as shown in Figure 3. Specifically, H^+^ can couple to generate H_2_ or undergo PCET with the *CO to generate *CHO (a key reaction step for CH_4_). In contrast, for multi-carbon products like C_2_H_4_, the RLS involves C-C coupling, which is less dependent on proton transfer and pH [69]. Notably, increasing the local pH helps reduce the overpotential of C-C coupling at RHE and enhance the selectivity for multi-carbon products [35]. Furthermore, both CH_4_ and C_2_H_4_ formation share the common *CO intermediate, while both CH_4_ and H_2_ formation involve the common *H, suggesting that the formation of C_1_ products is closely related to changes in pH [25,70]. Table 2 compares the FE of various CO_2_ reduction products in both alkaline and acidic electrolytes, encompassing both previous and recent advances. The comparative analysis demonstrates that product selectivity depends not only on catalyst composition but is also significantly influenced by key electrolyte parameters, particularly pH and ion effects (cations/anions). These electrolyte-mediated controls have enabled progressive improvements in CO_2_ conversion across different reaction pathways.

In summary, pH regulates proton availability and the reaction pathways of intermediates, determining the product distribution. Acidic electrolytes help prevent carbonate/bicarbonate formation but may promote HER. Alkaline electrolytes help suppress HER while leading to lower conversion efficiency. At the EEI, local pH changes can influence proton transfer and RLS, which controls the product reaction pathway. Consequently, optimizing pH can effectively enhance CO_2_RR efficiency, especially for multi-carbon products.

## 4. Cation Effects

**Figure 4:**
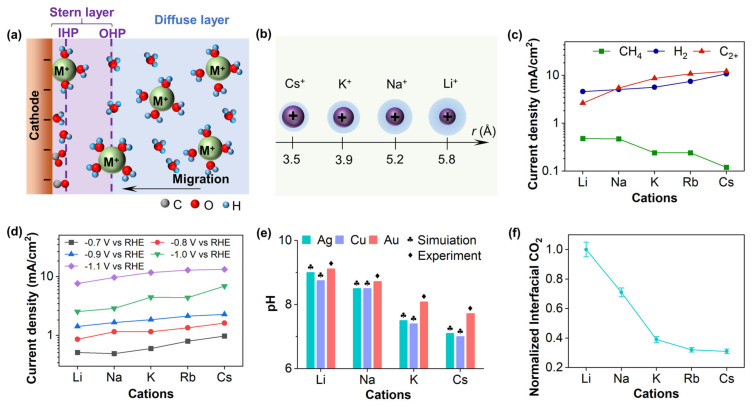
(**a**) Schematic diagram of the electric double layer. (**b**) The radius of the hydrated alkali metal cation [89]. (**c**) Current density on carbon paper supported Cu microparticles at −0.7 V [96]. Copyright 2020, American Association for the Advancement of Science. (**d**) Average current densities obtained during bulk electrolysis on Cu (100) as a function of metal cation at different potentials [93]. (**e**) Steady-state pH at different metal−electrolyte interfaces at −1 V vs. RHE [99]. (**f**) Tracking the interfacial CO_2_ concentration for the different alkali metal bicarbonate electrolytes by normalizing to the band area with Li [101]. All figures (**b**,**d**–**f**) are adapted from the American Chemical Society, Copyright 2017–2024.

Cations in the electrolyte are considered to be critical factors influencing the kinetics and product selectivity of electrocatalytic CO_2_RR, and their interactions with the electrode include specific adsorption and electrostatic adsorption (long-range interactions) [84,85]. According to the classical theory of the EDL, particularly the Gouy–Chapman–Stern (GCS) model [86,87], the specifically adsorbed species reside at the inner Helmholtz plane (IHP), while the electrostatically adsorbed species are located at the outer Helmholtz plane (OHP), as illustrated in Figure 4a. In the GCS model, there is no potential gradient along the planes parallel to the electrode surface, and the potential only varies in the direction perpendicular to the electrode surface. Murata et al. [88] first demonstrated that the activity and selectivity of CO_2_RR are influenced by AMCs, such as Li^+^, Na^+^, K^+^, Cs^+^, in the electrolyte on Cu catalysts. Based on the EDL structure, the larger the size of the AMCs (Li^+^ < Na^+^ < K^+^ < Cs^+^), the smaller the degree of hydration, with the hydration thickness following the order Cs^+^ + *n*H_2_O < K^+^ + *n*H_2_O < Na^+^ + *n*H_2_O < Li^+^ + *n*H_2_O [89], as shown in Figure 4b. Hydrated cations are generally adsorbed at the OHP rather than directly on the electrode surface, with their hydration shells interacting with the negative charges on the cathode [90,91,92]. Although various studies have been conducted on cations and proposed thorites to elucidate these effects, the cations mechanisms remain multifaceted. Typically, the role of cations in CO_2_RR can be categorized into three aspects: (1) modulating the interfacial electric field through non-covalent interactions; (2) controlling local CO_2_ concentration by buffering the interfacial pH; and (3) stabilizing intermediates through electric field–dipole interactions [92,93,94].

Non-covalent interactions, such as electrostatic interactions, lead cations to accumulate at the OHP, thereby altering the activity and selectivity of CO_2_RR [95]. The strength of the interfacial electric field (IEF) is the primary reason for the gradual increase in CO_2_RR reactivity from Li^+^ to Cs^+^ [96]. Figure 4c illustrates that, at −0.7 V, when the cation in the electrolyte is replaced from Li^+^ to Cs^+^, the current densities of C_2+_ and H_2_ significantly increase, while CH_4_ becomes a minor product for all cations. The trend of CO_2_RR reactivity in the presence of different AMCs is generally consistent with that in HCO_3_^−^ electrolytes reported by Resasco [93]. DFT calculations also confirm the value of the electrostatic stabilization. As shown in Figure 4d, during the electrochemical reduction of CO on Cu (100), the size of the AMCs affects the local current at the same potential. Similarly, the partial currents of HCOO^−^, C_2_H_4_, and C_2_H_5_OH generated on the Cu (111) surface also increase with the size of the cation. Nevertheless, Resasco suggest that the generation rates of H_2_ and CH_4_ are less influenced by cation size, which may be due to the absence of a dipole in the hydrogen ion or the presence of distinct counter-ions in the electrolyte (e.g., OH^−^ and HCO_3_^−^). Additionally, AMCs adsorbed at the OHP can suppress HER by altering the distribution of the IEF, limiting the migration of hydrated hydrogen ions to the cathode surface [97]. In comparison, the electric field of cations is more likely to stabilize CO rather than *CHO (the intermediate of CH_4_), which affects the formation of C_1_ products [93]. These trends are consistent with previous studies [92,98]. Hydrated cations with smaller sizes have larger surface charges and interfacial fields, thus requiring a smaller driving force for CO_2_RR at specific potentials. Therefore, cations are considered a necessary condition for promoting the CO_2_RR reaction.

Another theory posits that hydrated AMCs undergo hydrolysis reactions, acting as buffering agents to regulate the local pH and CO_2_ concentration at the EEI [92]. The O–H bonds within the hydration shells exhibit enhanced polarization through interactions with the negatively charged cathode, thereby facilitating the adsorption performance. This phenomenon shifts the OHP potential more negative, and increases the hydrogen ion concentration at the EEI and lower the local pH. In contrast to HER, a low-pH environment favors CO_2_RR, as the efficiency of CO_2_RR can be enhanced under elevated CO_2_ concentrations compared to HER, which is predominantly affected by a low pH [92]. Ayemoba et al. [99] and Zhang [100] independently determined the local pH of different AMCs during CO_2_RR using in-site surface-enhanced infrared absorption spectroscopy (SEIRAS) and rotating ring-disk electrode (RRDE) techniques, respectively. The results propose that the local pH follows the trend Li^+^ > Na^+^ > K^+^ > Cs^+^ (Figure 4e), consistent with conclusions of Murata [88]. However, direct experimental validation of the interfacial CO_2_ concentration trends under varying cations in CO_2_RR conditions remains lacking [101]. Contrary to the cation-buffering hypothesis, Malkani et al. [101] employed SEIRAS to probe the interfacial CO_2_ concentration dependence on cation size for a Au electrode under −0.8 V, revealing that larger AMCs correlate with lower interfacial CO_2_ concentrations (Figure 4f). Although the cation hydrolysis theory was developed based on Ag and Cu surfaces, which differ from Au in their potential of zero charge, the cation-buffering effect on interfacial CO_2_ concentration exhibits similar trends across Au, Ag, and Cu surfaces [99]. This study, combining reaction activity and spectroscopic results, demonstrates that interfacial CO_2_ concentration is primarily governed by reaction kinetics rather than cation-buffering capacity.

In the third theoretical framework, cations can influence CO_2_RR by inducing interactions between the electric field within the EDL and intermediates with large dipole moments, such as *CO_2_, *CO, and *OCCO. Specifically adsorbed AMCs at the electrode surface significantly modulate the binding strength or coverage of these intermediates [24,85]. Resasco et al. [93] propose that pronounced dipole interactions exist between adsorbed intermediates and hydrated cations, with interaction strengths following the order Cs^+^ > Rb^+^ > K^+^ > Na^+^ > Li^+^. According to density functional theory (DFT) calculations, this trend arises from the higher concentration of larger cations at the OHP. Further studies corroborate this theory; for instance, Ovalle et al. [102] utilized SEIRAS to investigate the displacement effects of tetramethylammonium (Me_4_N^+^) against AMCs, revealing an adsorption sequence of Li^+^ < Na^+^ < K^+^ < Cs^+^. Additionally, Monteiro et al. [103] demonstrated that CO_2_ cannot be reduced to CO on Au, Ag, or Cu electrodes in the absence of AMCs, thereby ruling out the influence of cation-mediated electric fields and pH buffering on CO_2_RR. Based on these studies, the authors propose three cation effects: (1) stabilizing intermediates via short- and medium-range interactions; (2) activating CO_2_ by reducing the O–C–O bond angle; and (3) enhancing electron transfer rates from the electrode surface to CO_2_. Furthermore, Monteiro et al. [31] explored the impact of cations with varying valences (e.g., Cs^+^, Ba^2^^+^, Nd^3^^+^) on CO_2_RR the HER. They argue that +3-valent cations have stronger acidity and higher hydrolysis propensity and can promote HER. Specifically, strongly acidic Nd^3^^+^ facilitates hydrolysis, leading to water reduction even at low overpotentials. In contrast, Cs^+^ and Ba^2^^+^ exhibit slower hydrolysis kinetics, thereby favoring CO_2_RR.

In summary, cation size significantly impacts CO_2_RR performance, with larger AMCs generally enhancing C_2+_ production while suppressing CH_4_. This occurs through three key mechanisms: (1) stronger interfacial electric fields that stabilize *CO intermediates, (2) local pH buffering that increases CO_2_ concentration, and (3) dipole interactions with key intermediates. Faradaic efficiency for C_2+_ products improves with cation size (up to ∼60% for Cs^^+^^), while current densities increase due to enhanced electric fields. However, trivalent cations (e.g., Nd^3^^+^) favor HER through acidic hydrolysis. These effects collectively demonstrate how cation selection can tune product selectivity.

## 5. Anion Effects

Anions, as an indispensable component of the electrolyte, are also crucial for CO_2_RR. Current research primarily focuses on specifically adsorbed anions, which chemically interact with the electrode substrate or undergo chemisorption with other electrolyte species. This interaction can dramatically alter reaction rates and selectivity by modulating the local pH at the EEI via buffering capacity, restructuring the catalyst surface, and affecting the adsorption/desorption of intermediates [85,104]. In the electrocatalytic process, anions are able to occupy active sites, leading to catalyst poisoning and hindering the adsorption of reactants or intermediates, thereby slowing down reaction kinetics [33]. Contrary to this conclusion, some studies have proposed that certain anions can enhance reaction kinetics, and the coverage of adsorbed anions should not be excessive [34,105].

**Figure 5:**
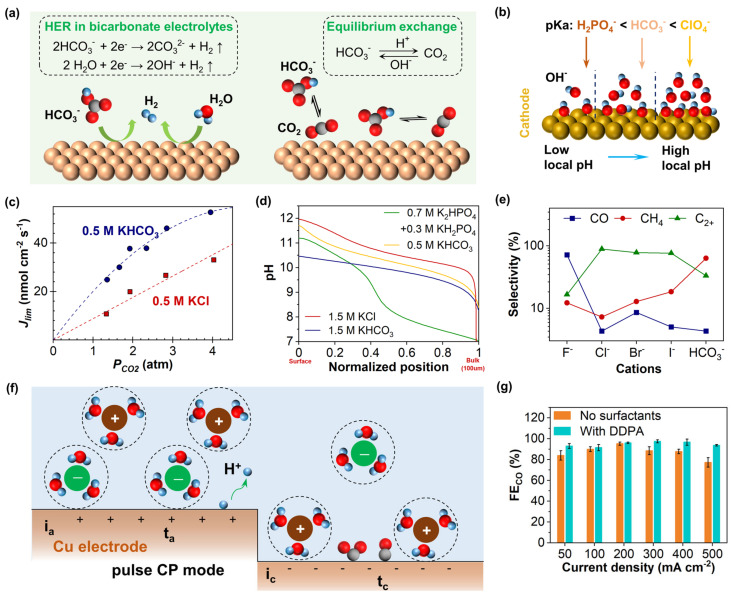
(**a**) Schematic diagram of the equilibrium conversion mechanism between CO_2_ and bicarbonate, and (**b**) the effect of the buffering capacity of anions on local pH [85]. Copyright 2024, John Wiley and Sons Ltd. (**c**) CO_2_ pressure (P_CO_2__) dependence of the limiting rate of mass transport of CO_2_ (*J_lim_*) in 0.5 M KHCO_3_ and 0.5 M KCl solutions, and (**d**) pH within the 100 μm boundary layer at P_CO_2__ of 2 atm [106]. (**e**) The selectivity of CO, CH_4_, and C_2_ as a function of anions, and (**f**) schematic illustrations for the structures of an electric double layer under pulsed CP mode [4]. (**g**) FE of CO at different potentials [117]. Copyright 2018, 2023, and 2024. American Chemical Society.

The protons transfer near the electrode generates a large amount of OH^−^. Acting as a proton donor, phosphate ions (H_2_PO_4_^−^) can neutralize OH^−^ to buffer the interfacial pH to maintain a low value [106,107]. In contrast, anions such as perchlorate (ClO_4_^−^), sulfate (SO_4_^2−^), and halides (e.g., Cl^−^, Br^−^, I^−^) may elevate the local pH due to the lack of effective neutralizing species, which inhibits the formation of certain products [108,109,110,111,112]. Dunwell et al. [59] proposed that most CO_2_(aq) in the electrolyte originates from the equilibrium with HCO_3_^−^ rather than the diffusion of CO_2_(g). In other words, HCO_3_^−^ serves as a carbon source to promote CO generation on Au electrodes, although it does not directly participate in the RLS of the CO formation [113]. Instead, it acts as a proton donor for both CO_2_RR and HER in the electrolyte, as shown in Figure 5a, complicating the role of HCO_3_^−^. Previous studies have compared the buffering effects of several anions on local pH using pKa (Figure 5b), revealing the following order of pH increase: H_2_PO_4_^−^ < HCO_3_^−^ < ClO_4_^−^. Although ClO_4_^−^ can suppress HER, the slower kinetics result in a lower selectivity at higher potentials [114]. As a supplement, the buffering capacity of KHCO_3_, KCl and phosphate electrolytes on the CO_2_RR rate and local pH were explored [106]. Under CO_2_-limiting conditions, the CO_2_ consumption rate (J_lim_) in KHCO_3_ solutions is notably higher than that in KCl solutions (Figure 5c). Specifically, at higher CO_2_ pressures (P_CO_2__), J_lim_ exhibits nearly linear growth in KCl solutions, whereas it exhibits a nonlinear increase in KHCO_3_ solutions. Consequently, the CO_2_RR is not only influenced by KHCO_3_ concentration but is also closely related to its excellent buffering capacity. The simulation results in Figure 5d further demonstrate that KHCO_3_ can maintain a relatively stable pH at the EEI, preventing excessive alkalinity in the local environment. On the contrary, KCl electrolytes lack buffering capacity, causing a pronounced pH increase at the electrode surface.

Halide ions (e.g., Br^−^, I^−^, Cl^−^) can promote CO_2_RR, especially on Cu electrodes. On Cu (100) surfaces, halide ions enhance the FE of C_2_ products while reducing H_2_ [111]. At −1.23 V, the FE of C_2_H_4_ in KI electrolyte reaches 50.3%, which is higher than the 30.6% observed in the KClO_4_ electrolyte. Similarly, the FE of C_2_H_5_OH increases from 7.1% to 16.4%, while that of CO rises from 11.8% to 22.8%. These results indicate that I^−^ can alter the electronic environment of *CO, enhancing its adsorption strength and surface coverage, and effectively lowering the energy barrier for C–C coupling. Also, halide ions are crucial in restructuring the catalyst surface, leading to the formation of highly rough surfaces that provide more active sites [34]. For instance, Garg et al. [115] investigated the effects of different halide ions in choline-based electrolytes on reducing CO_2_ to CO on the Ag electrode. They illustrated that the FE of CO follows the order Cl^−^ > Br^−^ > I^−^. At more negative potentials, halide ions promote the dissolution and redeposition of Ag electrodes, forming high-index crystal facets, such as (220), (311) (222). Beyond restructuring catalyst surface, halide ions also modulate the formation of key intermediates in the reaction pathway. Their charge-enabling properties facilitate the formation of *COOH, not only reducing the overpotential but also increasing the number of adsorbed CO species available for coupling [111,116]. Wang et al. [4] studied the effects of three different anions (F^−^, Cl^−^, HCO_3_^−^) on CO_2_RR and proposed an anion enrichment strategy to regulate ion adsorption and desorption. By periodically applying positive potentials to the cathode during pulsed electrolysis, anions can be adsorbed in the IHP, increasing the local anion concentration (Figure 5f). The results show that KF, KCl, and KHCO_3_ electrolytes exhibit the highest selectivity for CO, C_2+_ and CH_4_, respectively, as illustrated in Figure 5e. The strong electronegativity of F^−^ enables it to strongly adsorb on the electrode surface, inhibiting further reduction of *CO. The moderate adsorption strength of Cl^−^ favors C–C coupling between *CO species. For HCO_3_^−^, its strong proton-donating ability promotes the hydrogenation of *CO, enhancing CH_4_ selectivity. A key advantage of this strategy is that pulsed electrolysis periodically pushes protons away from the electrode surface, reducing the proton source and significantly suppressing HER.

Organic anions primarily influence CO_2_RR by restructuring the electrode surface. For instance, under −0.8 V vs. RHE, propionate (C_3_H_5_CO_2_^−^) increases the FE of CO to 98.7%, far exceeding the 80% achieved with HCO_3_^−^ [114]. Molecular dynamics simulations reveal that carboxylate form a suitable interfacial water structure through weak adsorption on the electrode surface, promoting CO_2_ reduction while inhibiting HER. Additionally, Ge et al. [110] investigated the effects of different anionic surfactants, including sodium dodecylbenzene sulfonate (SDS), sodium lauryl sulfate (SLS), sodium monolauryl phosphate (SMP), and sodium laurate (SL), in KHCO_3_ electrolyte. They found that these additives significantly improved the FE of CO at −1.2 V vs. RHE, reaching 89.7%, 97.5%, 98.4%, and 98.9%, respectively, far exceeding the 53.1% FE observed in the absence of surfactants. Simultaneously, the FE of H_2_ significantly decreased. These results demonstrate that surfactants not only enhance CO selectivity but also suppress HER. In-site attenuated total reflection surface-enhanced infrared spectroscopy (ATR-SEIRAS) analysis revealed that surfactants strengthen the H-bond network of interfacial water molecules, promoting proton-coupled reactions and inhibiting HER. DFT calculations further support these anions in improving water structure, showing that SL and SMP exhibit a stronger H-bond than SDS and SLS. The organic compound dodecyl phosphate (DDPA) also can restructure the H-bond network at the EEI, increasing the proportion of free water [117]. DDPA increases the FE of CO from 70% to 98% at −1.0 V vs. RHE, maintaining over 90% efficiency for 8 h in flow electrolysis, demonstrating a significant enhancement in CO_2_ reduction performance.

In summary, anions have remarkable effects on CO_2_RR performance through multiple mechanisms: (1) pH regulation via buffering capacity (e.g., H_2_PO_4_^−^ maintains a low pH while ClO_4_^−^ elevates it), (2) surface restructuring (halides create rough surfaces and high-index facets), and (3) intermediate stabilization (I^−^ enhances *CO adsorption for C–C coupling). These effects lead to distinct product distributions—halides boost C_2_+ Faradaic efficiency, while F^−^ favors CO and HCO_3_^−^ promotes CH_4_. Organic anions like carboxylates and surfactants further enhance CO selectivity (up to 98.9% FE) by optimizing interfacial water structure. Current density variations arise from altered reaction kinetics, with buffering anions sustaining higher CO_2_ consumption rates than non-buffering ones. The interplay between anion-specific adsorption strength and proton management ultimately dictates reaction pathway length, with moderately adsorbing species favoring multi-carbon products while strongly adsorbing ones that terminate at CO.

## 6. Summary and Outlook

This review summarizes the critical role of the electrolyte in electrochemical CO_2_RR, including the effects of pH, cations, and anions on reaction pathways, activity, and selectivity. The research shows the following findings:

1. The configuration of reaction intermediates significantly influences the product formation, and exploring the universality of pathways remains a key focus.
2. The local pH of the electrolyte not only affects the source of protons but also regulates intermediates.
3. Cations significantly affect the kinetics and selectivity of CO_2_RR through non-covalent interactions, buffering the interface pH, and stabilizing intermediates.
4. Anions alter the reaction rate and product distribution by regulating local pH, catalyst surface reconstruction, and the adsorption/desorption processes of intermediates.

Although numerous studies have highlighted the critical role of the electrolyte environment in CO_2_RR, several key challenges and unresolved questions remain. Addressing these challenges will be essential for advancing the field and improving the efficiency and selectivity of CO_2_RR.

1. The interaction mechanisms between the effects of cations and anions are not yet fully understood, particularly in complex electrolyte systems, making it difficult to isolate and analyze individual contributions.
2. The stability of CO_2_RR in acidic electrolytes remains a significant issue, as catalyst dissolution and dynamic changes in the local microenvironment require further investigation.
3. The formation pathways of multi-carbon products are intricate, necessitating a combination of advanced experimental techniques and theoretical calculations to elucidate the underlying reaction mechanisms.

## Figures and Tables

**Table 1:** The reactions of CO_2_RR and HER [18]. Copyright 2019, American Chemical Society.

Reaction	Potential (V vs. RHE)
*x* CO_2_ + *n* H^+^ + *n* e^−^→*product* + *y* H_2_O	
CO_2_ + 2H^+^ + 2e^−^→CO (g) + H_2_O	−0.10
CO_2_ + 2H^+^ + 2e^−^→HCOOH (aq)	−0.12
CO_2_ + 4H^+^ + 4e^−^→C (s) + 2H_2_O	0.21
CO_2_ + 6H^+^ + 6e^−^→CH_3_OH (aq) + H_2_O	0.03
CO_2_ + 8H^+^ + 8e^−^→CH_4_ (aq) + 2H_2_O	0.17
2CO_2_ + 8H^+^ + 8e^−^→CH_3_COOH (aq) + 2H_2_O	0.11
2CO_2_ + 10H^+^ + 10e^−^→CH_3_CHO (aq) + 3H_2_O	0.06
2CO_2_ + 12H^+^ + 12e^−^→C_2_H_4_ (q) + 4H_2_O	0.08
2CO_2_ + 12H^+^ + 12e^−^→C_2_H_5_COOH (aq) + 3H_2_O	0.09
2CO_2_ + 14H^+^ + 14e^−^→C_2_H_6_ (g)+ 4H_2_O	0.14
3CO_2_ + 16H^+^ + 16e^−^→C_2_H_5_CHO (aq) + 5H_2_O	0.09
3CO_2_ + 18H^+^ + 18e^−^→C_3_H_7_OH (aq) + 5H_2_O	0.10
H_3_O^+^→H^+^ + H_2_O	-
2H^+^ + 2e^−^→H_2_	0
2H_2_O + 2e^−^→H_2_ + 2OH^−^	-

**Table 2:** Comparative analysis of electrolyte effects in CO_2_RR.

Parameters	Previous Studies	Recent Advances
CO FE	~80% (H_2_SO_4_ + Cs_2_SO_4_, Ag) [71]	95% (K_2_SO_4_, Ag@C) [65] 95% (H_2_SO_4_, c-PDDA-Ag) [72] 97.1% (KHCO_3_, Fe_2_C-Cs@DC) [73]
HCOOH FE	80% (KHCO_3_, In NCs) [74] 89.2% (Na_2_SO_4_, Porous Bi) [75]	90.15% (KOH, BOC/Bi-3) [76] 90.8% (K_2_SO_4_ + H_2_SO_4_, Sn-SAC) [77] 93% (K_2_SO_4_, r-Pb) [77]
CH_4_ FE	~57% (KOH, La_2−x_ CuO_4−δ_) [78]	80% (DMSO, Cu) [79] 71% (H_2_SO_4_, EDTA-Cu) [80]
C_2_H_4_ FE	26% (H_3_PO_4_ + KCl, CAL-Cu) [24] ~63% (KOH, CuO-160W) [81]	70% (K_2_SO_4_ + H_2_SO_4_, C/Cu/PTFE) [82] 74% (KOH, Dendritic CuO) [83]

## Data Availability

The data that support the findings of this study are available from the corresponding authors upon reasonable request.
